# Lsr2, a pleiotropic regulator at the core of the infectious strategy of *Mycobacterium abscessus*

**DOI:** 10.1128/spectrum.03528-23

**Published:** 2024-02-14

**Authors:** Elias Gerges, María del Pilar Rodríguez-Ordoñez, Nicolas Durand, Jean-Louis Herrmann, Frédéric Crémazy

**Affiliations:** 1 Université Paris-Saclay, UVSQ, Inserm, Infection et inflammation, Montigny-Le-Bretonneux, France; 2 Université Paris-Saclay, Université d’Evry, Laboratoire Européen de Recherche pour la Polyarthrite rhumatoïde-Genhotel, Evry, France; 3 APHP, GHU Paris-Saclay, Hôpital Raymond Poincaré, Service de Microbiologie, Garches, France; CNRS - University of Toulouse, Toulouse, France

**Keywords:** *Mycobacterium abscessus*, S/R morphotypes, Lsr2, nucleoid-associated protein, transcription factor, antibiotic resistance

## Abstract

*Mycobacterium abscessus* is a non-tuberculous mycobacterium, causing lung infections in cystic fibrosis patients. During pulmonary infection, *M. abscessus* switches from smooth (Mabs-S) to rough (Mabs-R) morphotypes, the latter being hyper-virulent. Previously, we isolated the *lsr2* gene as differentially expressed during S-to-R transition. *lsr2* encodes a pleiotropic transcription factor that falls under the superfamily of nucleoid-associated proteins. Here, we used two functional genomic methods, RNA-seq and chromatin immunoprecipitation-sequencing (ChIP-seq), to elucidate the molecular role of Lsr2 in the pathobiology of *M. abscessus*. Transcriptomic analysis shows that Lsr2 differentially regulates gene expression across both morphotypes, most of which are involved in several key cellular processes of *M. abscessus*, including host adaptation and antibiotic resistance. These results were confirmed through quantitative real-time PCR, as well as by minimum inhibitory concentration tests and infection tests on macrophages in the presence of antibiotics. ChIP-seq analysis revealed that Lsr2 extensively binds the *M. abscessus* genome at AT-rich sequences and appears to form long domains that participate in the repression of its target genes. Unexpectedly, the genomic distribution of Lsr2 revealed no distinctions between Mabs-S and Mabs-R, implying more intricate mechanisms at play for achieving target selectivity.

**IMPORTANCE:**

Lsr2 is a crucial transcription factor and chromosome organizer involved in intracellular growth and virulence in the smooth and rough morphotypes of *Mycobacterium abscessus*. Using RNA-seq and chromatin immunoprecipitation-sequencing (ChIP-seq), we investigated the molecular role of Lsr2 in gene expression regulation along with its distribution on *M. abscessus* genome. Our study demonstrates the pleiotropic regulatory role of Lsr2, regulating the expression of many genes coordinating essential cellular and molecular processes in both morphotypes. In addition, we have elucidated the role of Lsr2 in antibiotic resistance both *in vitro* and *in vivo*, where *lsr2* mutant strains display heightened sensitivity to antibiotics. Through ChIP-seq, we reported the widespread distribution of Lsr2 on *M. abscessus* genome, revealing a direct repressive effect due to its extensive binding on promoters or coding sequences of its targets. This study unveils the significant regulatory role of Lsr2, intricately intertwined with its function in shaping the organization of the *M. abscessus* genome.

## INTRODUCTION

*Mycobacterium abscessus* is a rapidly growing mycobacterium that has emerged as a significant pathogen in humans (1, 2). This species is known to be one of the most pathogenic non-tuberculous mycobacteria (NTM), representing a serious threat to the health and well-being of cystic fibrosis patients (3, 4). In comparison to other NTMs, *M. abscessus* infections have been found to have particularly deleterious effects on ventilatory functions (5). It is also responsible for persistent chronic infections associated with the formation of inflammatory granulomas (6). Furthermore, analysis of the *M. abscessus* genome has revealed the presence of several genes associated with multi-resistance to antibiotics and disinfectants, which make it hard to treat and eradicate (7–9).

Like other mycobacteria, *M. abscessus* displays two distinct morphotypes phenotypically different in their motility and their ability to grow biofilm: smooth (Mabs-S) and rough (Mabs-R) colony morphotypes (10, 11). The loss of surface-associated glycopeptidolipids (GPL) stands as the hallmark of the Mabs-R morphotype, serving as a critical element in its pathogenic strategy (11, 12). Indeed, the transition from Mabs-S to Mabs-R has been found to be correlated with a significant increase in pathogenicity and modulation of the immune system response (13, 14). Additionally, Mabs-R is associated with increased apoptosis and autophagy in cells, leading to cell death and the formation of aggregates or serpentine cords in the extracellular environment, facilitating the multiplication of the bacteria (15, 16). Thus, understanding the mechanisms behind S-to-R transition that only occurs during pulmonary infections is key for unraveling the pathogenicity of *M. abscessus*. Genomic analysis comparing couples of isogenic morphotypes of Mabs-S and Mabs-R identified several insertions/deletions (indels) and single-nucleotide polymorphism that impair the correct expression of genes involved in the synthesis (*mps1* and *mps2*) and the transport (*mmpL4b*) of GPL (17). Furthermore, we also identified the gene encoding the leprosy serum reactive clone 2 (Lsr2) protein as exhibiting significantly higher expression level in Mabs-R, thereby indicating its involvement in the heightened pathogenicity of this morphotype.

Originally identified as an immunodominant antigen in *Mycobacterium leprae* (18), Lsr2 has subsequently been isolated in other mycobacteria (19, 20) and *Streptomyces* (21). This 12.4-kDa small basic protein belongs to the nucleoid-associated proteins (NAPs), a well-known family of chromosomal organizers and global transcription factors (22). Recent studies have highlighted the pivotal role of NAPs as major contributors to bacterial adaptation in response to changes in environmental conditions and stress, particularly during host infections (23). Lsr2 acts as a functional ortholog of the heat-stable nucleoid-structuring protein (H-NS), sharing its ability to preferentially bind AT-rich sequences, possibly forming rigid oligomers and bridging distant DNA fragments across the genome (24). Lsr2 functions as a pleiotropic regulator, influencing the expression of numerous genes involved in virulence and host-induced stress response (25). Chromatin immunoprecipitation-sequencing (ChIP-seq) studies performed in *Mycobacterium tuberculosis* have revealed that Lsr2 binds to genes associated with early antigenic secretion (ESX) systems, the biosynthesis of phthiocerol dimycocerosate and phenolic glycolipid cell wall lipids, and encoding antigenic proteins of the proline-glutamate (PE) and proline-proline-glutamate (PPE) family (20). Additionally, Lsr2 has been shown as crucial for *M. tuberculosis’* ability to vary oxygen levels and protect bacilli during macrophage infection against reactive oxygen intermediates (ROI) (26, 27). Unlike in *M. tuberculosis*, Lsr2 is not vital for *Mycobacterium smegmatis* survival but impacts colony morphology, biofilm formation, and adaptation to hypoxia and antibiotics (19, 28).

We recently showed that in *M. abscessus*, Lsr2 is crucial for intracellular growth of both morphotypes within macrophages and amoeba (29). Deletion of *lsr2* increases the susceptibility of the Mabs-R to oxidative species, highlighting the role of Lsr2 in protecting DNA integrity from ROI during macrophage infection. Furthermore, Lsr2 is essential for the virulence of *M. abscessus* in the zebrafish model and persistence in the lungs of infected mice. Here, we used an integrated functional genomic approach to delve into the function of Lsr2 in both S and R morphotypes of *M. abscessus*. Moreover, we investigated its impact on host adaptation and its contribution to antibiotic resistance.

## RESULTS

### Differential expression analysis of the Mabs-S and Mabs-R transcriptomes of *M. abscessus*

We first confirmed our precedent results by performing RNA-seq and comparing gene expression on the S/R pair of 19977-IP (CIP) wild-type (WT) control strains. As shown in a prior study conducted by Pawlik and co-workers (17)*,* our data revealed only a handful of genes showing differential expression between the two morphotypes. Specifically, we observed that four genes were more expressed in Mabs-S, while nine genes were more expressed in Mabs-R (Table S2). Notably, among these differentially expressed genes, we identified key players in the S/R transition, such as the genes of GPL locus (*mps1, mps2,* and *gap*). Our current RNA-seq analysis also unveiled decreased expression of GPL locus genes in Mabs-R compared to Mabs-S. This finding aligns with our previous study, which reported the absence of mRNA transcripts for the *mps1-mps2-gap* operon in the Mabs-R due to the CG insertion in the 5*'* region of *mps1*. Furthermore, both our work and the aforementioned study highlight differential regulation of the genes encoding the Nrd family proteins between the two morphotypes. Specifically, transcript expression levels of *nrdF, nrdE, nrdI,* and *nrdH* were significantly increased in Mabs-R. These genes are known to be involved in the nucleotide metabolism pathway as well as in the persistence of mycobacteria within phagolysosomes.

### Differential gene expression analysis of wild-type and *lsr2* mutant strains for Mabs-S and Mabs-R

**Fig 1:**
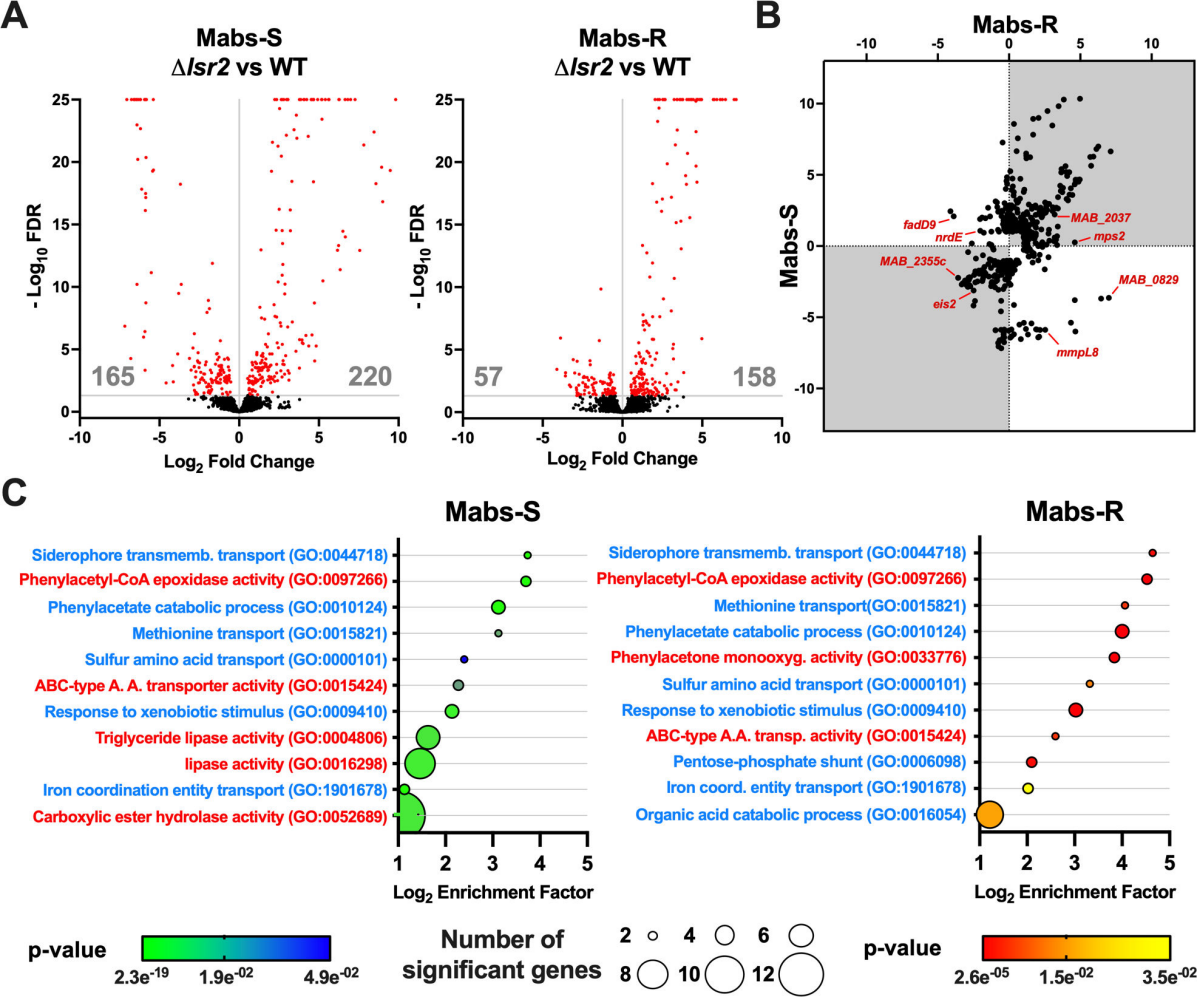
Analysis of Lsr2 regulon in *M. abscessus* morphotypes. (**A**) Volcano plots showing differentially expressed genes (DEG) (red) in Mabs-S-Δ*lsr2* vs Mabs-S-WT (left) and Mabs-R-Δ*lsr2* vs Mabs-R-WT (right). Genes exhibiting a greater than two fold changes in expression between two conditions [log_2_ fold change (log_2_FC) ≤−1 or ≥1] and with a false discovery rate (FDR) of less than 0.05 are considered significantly deregulated. Genes with log_2_FC ≥1 correspond to downregulated genes by Lsr2 (repressed genes), and genes with log_2_FC ≤−1 correspond to upregulated genes by Lsr2 (activated genes). (**B**) Plot representation showing all genes regulated by Lsr2 at least in one of the two morphotypes of *M. abscessus* with a log_2_FC ≥1 and log_2_FC ≤−1 of gene expression between the Δ*lsr2*-mutant strains and wild-type strains. (**C**) Gene ontology (GO) enrichment analysis was performed with the topGO R package. Enriched GOs (Biological Process in blue and Molecular Function in red) are sorted according to their enrichment factor, corresponding to the ratio of significant DEGs assigned to the GO over expected assigned DEGs to the GO as defined by topGO. Enriched GOs are represented by circles whose size is proportional to the amount of significant DEGs assigned. Positive statistical tests are given that face each GO.

We performed differential gene expression analysis between *lsr2* mutant and wild-type strains to identify the Lsr2 targets in *M. abscessus*. Our RNA-sequencing study revealed that a total of 215 genes were regulated by Lsr2 in Mabs-R and 385 genes in Mabs-S. Interestingly, more than 50% of these genes were upregulated in both morphotypes (158 genes in Mabs-R and 220 genes in Mabs-S) in the *lsr2* mutant, providing further evidence for the repressor role of Lsr2 under optimal growth conditions (Fig. 1A). Among those, only 126 genes were common to both Mabs-S and Mabs-R (Fig. 1B). These include not only genes that are either repressed (e.g., *MAB_2037, MAB_2275, MAB_4467*) or activated in both morphotypes (e.g., *eis2, paaK, MAB_2355c*), but also genes that show opposite regulation (e.g., *mmpL8, fadD9, MAB_0829*). We also show that 67.2% and 41.4% of the deregulated genes are only regulated in Mabs-S and Mabs-R, respectively (e.g., *mps2, MAB_1012c, ispe*). This global analysis suggests a different regulatory role for Lsr2 in the two morphotypes depending on their physiologies.

Gene ontology (GO) analysis performed on both Mabs-S and Mabs-R showed that Lsr2 affects multiple categories of biological and functional processes in *M. abscessus*, confirming its role as a pleiotropic transcription regulator (Fig. 1C). Notably, Lsr2 exhibits a significant impact on the transport of different substrates in both morphotypes including siderophore transport (GO:0044718), methionine transport (GO:0015821), sulfur amino acid transport (GO:0000101), iron coordination entity transport (GO:1901678), and ABC-type amino acid transporter activity (GO:0015424). Pertaining to metabolism activity, we observed GO enrichment associated with phenylacetate catabolism (GO:0097266, GO:0010124, GO:0033776), metabolism of glucose (GO:0006098), and organic acid catabolism (GO:0016054). Interestingly, we observed an enrichment in lipid metabolism (GO:0016298, GO:0004806) exclusively in Mabs-S, suggesting a regulatory role for this protein based on the membrane lipid composition of the two morphotypes. In addition, GO related to bacterial stress response is also enriched in both Mabs-S and Mabs-R, particularly in the context of the xenobiotic stimulus (GO:0009410).

### Lsr2 modulates the expression of genes involved in intracellular growth and response to stress

**Fig 2:**
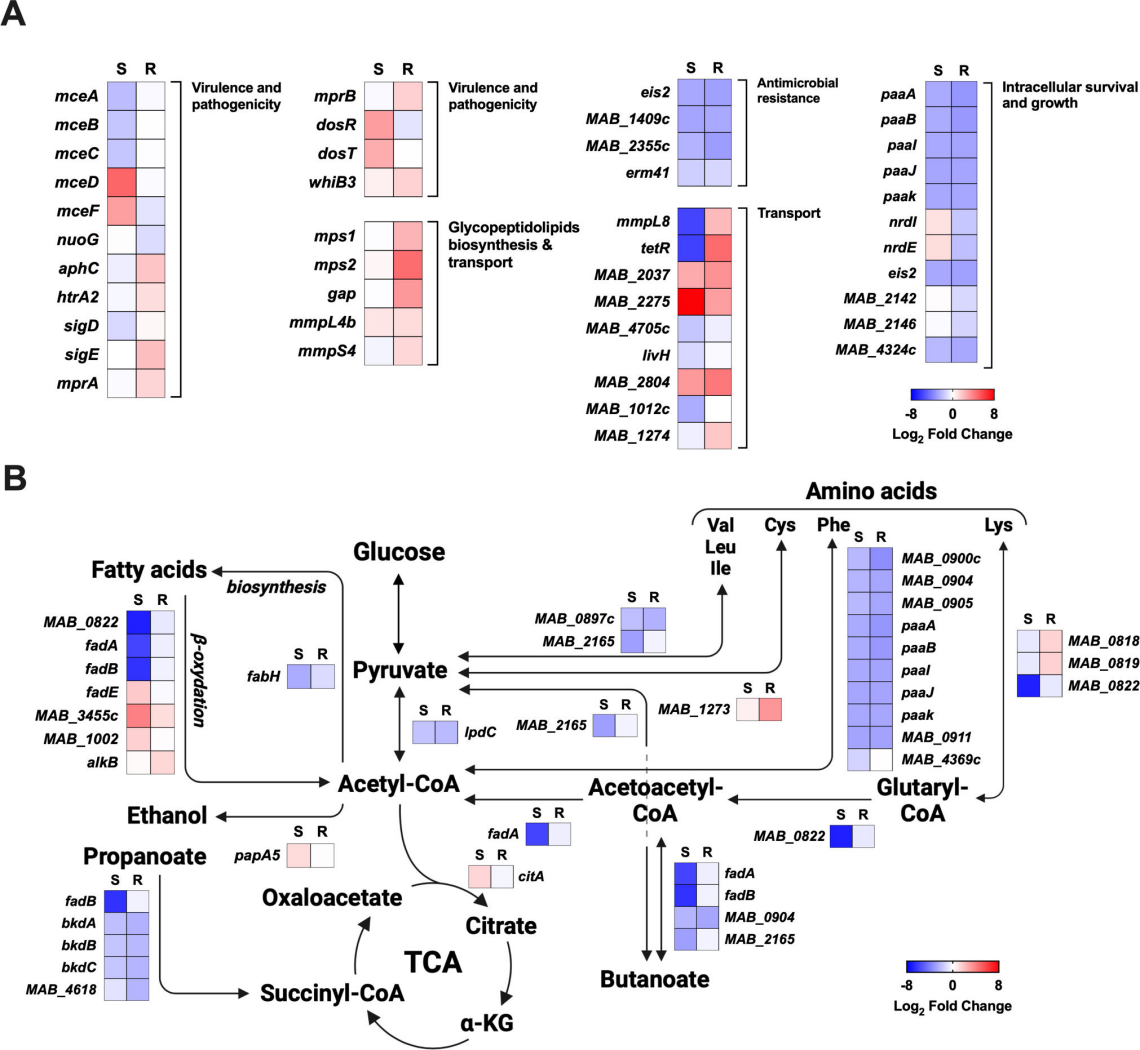
Lsr2 regulated genes involved in different pathways in *M. abscessus*. (**A and B**) Heat maps representing genes regulated by Lsr2 and implicated in various cellular mechanisms (intracellular survival and growth, virulence and pathogenicity, glycopeptidolipids biosynthesis, transport, antimicrobial resistance genes, and central carbon metabolism) in Mabs-S and Mabs-R. The color scale represents differentially expressed genes with a log_2_ fold change (log_2_FC) ranging from −8 to 8. Genes with log_2_FC ≥1 correspond to downregulated genes by Lsr2 (repressed genes), while genes with log_2_FC ≤−1 correspond to upregulated genes by Lsr2 (activated genes).

Our transcriptomic analysis shows that Lsr2 acts as a pleiotropic transcription factor in both morphotypes of *M. abscessus,* regulating a wide range of genes involved in key physiological and cellular processes. Lsr2 positively controls the expression of genes related to growth and intracellular survival (Fig. 2A), such as *paaA, paaB, paaI, paaJ,* and *paak*, which were acquired through horizontal transfer from *β-proteobacteria* and involved in phenylacetic acid degradation (7). *nrdI* and *nrdE*, two key players of intracellular survival (17), are both upregulated by Lsr2 in Mabs-R, while they are downregulated in Mabs-S. Deletion of *lsr2* leads to a significant decrease in the expression of *MAB_4532*c (*eis2*) that encodes the Eis N-acetyl transferase protein (30) both in Mabs-S and Mabs-R (Fig. 2A).

Additionally, we identified two genes, *MAB_2142* and *MAB_2146,* activated only in Mabs-R by Lsr2 and encoding [4F-4S] Ferredoxin proteins that function as intracellular stress sensors and contribute to genomic stability during macrophage and amoeba infections (30). Deletion of *lsr2* in Mabs-S and Mabs-R also results in a significant decrease in the expression of the *MAB_4324*c gene, which encodes an N-acetyltransferase recently found to enhance internalization and intracellular growth of mycobacteria in human macrophages (31) (Fig. 2A).

### Lsr2 plays a role in virulence and pathogenicity

Lsr2 modulates the expression of *mce* (mammalian cell entry) genes encoding proteins involved in virulence and pathogenicity through host cell recognition and invasion in *M. tuberculosis* (32, 33). For example, *mceA, mceB,* and *mceC* were more expressed in wild-type Mabs-S compared to Mabs-S-Δ*lsr2,* while other *mce* genes such as *mceD* and *mceF,* located in a different locus, were repressed (Fig. 2A). *nuoG, aphC,* and *htrA2*, all regulated in Mabs-R, impede macrophage antimicrobial effectors through their response to oxidative and nitrosative stress as well as their inhibitory effect on cell apoptosis and phagosomal maturation (34, 35). Interestingly, several families of transcriptional regulators playing a key role in virulence were targets of Lsr2. For example, *sigD* and *sigE* that code for two sigma factors regulated by Lsr2 in Mabs-S and Mabs-R, respectively, are involved in macrophage viability through resistance to oxidative stress (36, 37). Moreover, *mprA* and *mprB,* also regulated by Lsr2 in Mabs-R, are *sigD-*dependent transcriptional regulators involved in stress response pathways in *M. tuberculosis* (38). *dosR* and *dosT,* encoding essential factors for hypoxic adaptation, persistence, and virulence in *M. tuberculosis*, are tightly controlled by Lsr2 in Mabs-S (39, 40). Finally, Lsr2 controls *whiB3* transcription in Mabs-R, which is known to be involved in intraphagosomal stress resistance in *M. tuberculosis* (41, 42) and redox sensing in *M. smegmatis* (43). The transcriptomic results reveal a diverse range of Lsr2 targets involved in virulence, strongly suggesting its role in the infection, pathogenicity, and persistence of *M. abscessus*.

### Lsr2 regulates families of proteins involved in transport in *M. abscessus*

**Fig 3:**
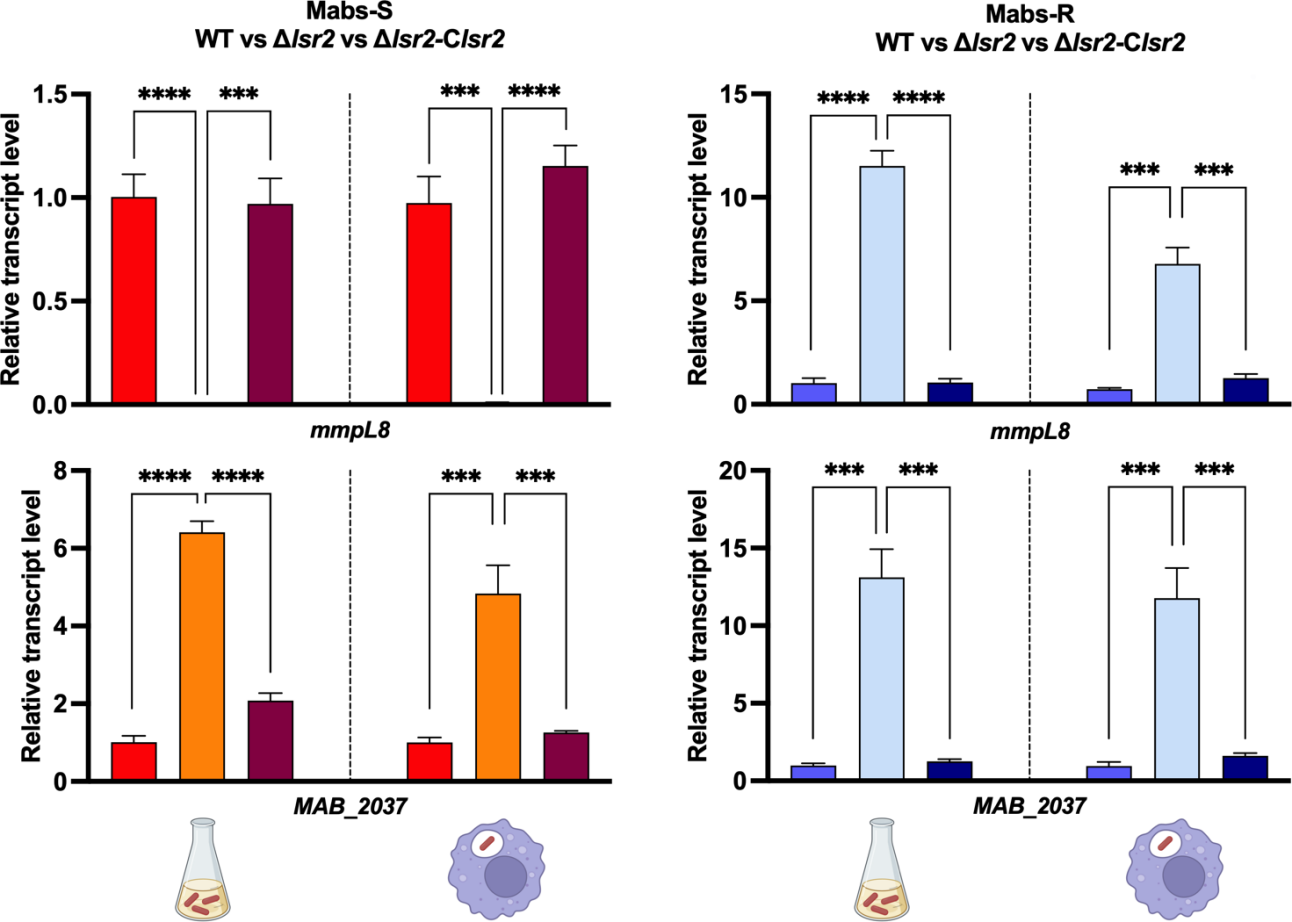
Conservation of Lsr2 regulatory effect on transport genes in both planktonic growth and intracellular growth conditions. Quantification of relative expression of *mmpL8* and *MAB_2037* measured by RT-qPCR under planktonic growth conditions (left side) and intracellular growth conditions (right side) in Mabs-S-WT (red), Mabs-S-Δ*lsr2* (orange), Mabs-S-Δ*lsr2*-C*lsr2* (purple), Mabs-R-WT (blue), Mabs-R-Δ*lsr2* (light blue), and Mabs-R-Δ*lsr2*-C*lsr2* (dark blue). *sigA* transcript levels were used for normalization. For each assay, *n* = 3, and error bars are SEM. ***P < 0.001 and ****P < 0.0001 (Student’s *t* test).

MmpL proteins (mycobacterial membrane protein large) play a crucial role in mycobacterial physiology by facilitating the export of lipid components, toxins, and metabolites across the cell envelope, as well as expelling antibiotics (44, 45). Interestingly, Lsr2 exhibits distinct regulation of the *MAB_0855* gene encoding the MmpL8 protein in both Mabs-S and Mabs-R (Fig. 2A; Table S3). MmpL8 was found to be essential for intracellular growth of *M. abscessus* in amoeba and macrophages. Its deletion significantly reduces virulence in zebrafish and disrupts interactions with macrophages, hampering the establishment of cytosolic connections (46). In Mabs-S, Lsr2 upregulated the *mmpL8* gene by around 56-fold compared to Mabs-S-Δ*lsr2*, while in Mabs-R, Lsr2 downregulated the *mmpL8* gene by approximately 5-fold in Mabs-R-Δ*lsr2*. To confirm whether Lsr2 exhibits the same regulatory effect *in vivo*, we performed quantitative real-time PCR (RT-qPCR) analysis on *mmpL8* using mRNA isolated from wild-type and *lsr2* mutants Mabs-S and Mabs-R morphotypes 16 hours after murine J774.2 macrophage infections. Consistent with transcriptomic analysis in planktonic cultures, Lsr2 regulation of the *mmpL8* gene persists during macrophage infections. We observed a significant increase in *mmpL8* transcripts in Mabs-R-Δ*lsr2*, while the expression of this gene was not detectable in Mabs-S-Δ*lsr2* (Fig. 3). This can also suggest that Lsr2 controls the expression of the *tetR* transcription factor, which in turn regulates the entire *mmpL8_MAB_* locus (Fig. 2A; Table S3). Additional examples for strongly regulated *mmpL* genes in Mabs-S and Mabs-R were *MAB_ 2037* and *MAB_2075*. The *MAB_2037* gene, belonging to mycolate synthesis operon (30), was significantly repressed by Lsr2 both in Mabs-S and Mabs-R (Fig. 2A). Again, the repressive effect of Lsr2 on the *MAB_2037* gene was confirmed by RT-qPCR after infection by both morphotypes of *M. abscessus* (Fig. 3). For all these genes, complementing the mutant strains with *lsr2* restored the same level of expression as observed in wild-type strains.

### *lsr2* deletion results in increased GPL locus transcripts in Mabs-R morphotype

The loss of GPL at the plasma membrane of *M. abscessus* is known as the hallmark of the S-to-R transition, and the role of Lsr2 as a negative regulator of GPL production was already described in *M. smegmatis* (47). However, our previous results showed no effect of Lsr2 deletion on either the GPL profile or the morphology of the bacterium (29). Upon deletion of *lsr2*, genes involved in GPL biosynthesis and transport (*mps1*, *mps2*, *gap,* and *mmpL4b*) showed increased expression only in Mabs-R, coinciding with the restoration of a full-length transcript, despite the presence of the genetic lesion, while no significant difference in Mabs-S was observed (Fig. 2A).

### Lsr2 is implicated in central carbon metabolism regulation

Through KEGG analysis, we investigated the impact of Lsr2 on various metabolic pathways in *M. abscessus* (Fig. 2B). Notably, our findings revealed that Lsr2 predominantly affects the pathways of fatty acid degradation (β-oxidation) and amino acid degradation in both morphotypes. Lsr2 exhibits both upregulation and downregulation of numerous genes encoding enzymes involved in β-oxidation, including *fad* genes encoding acyl-CoA synthetases, ultimately leading to acetyl-CoA production for further incorporation into the tricarboxylic acid cycle (Fig. 2B). The regulatory influence of Lsr2 extends to amino acid metabolism, particularly affecting the *paa* genes involved in intracellular growth, which exhibit increased expression in both morphotypes. Genes involved in propanoate metabolism were mostly highly expressed following *lsr2* deletion in Mabs-S and Mabs-R*,* including *bkdA*, *bkdB*, *bkdC,* and *MAB_4618* that facilitate the conversion of propionyl-CoA derived by β-oxidation of odd-chain fatty acids into succinate.

### Lsr2 controls antimicrobial resistance genes transcription and susceptibility to antibiotics in *M. abscessus*

**Fig 4:**
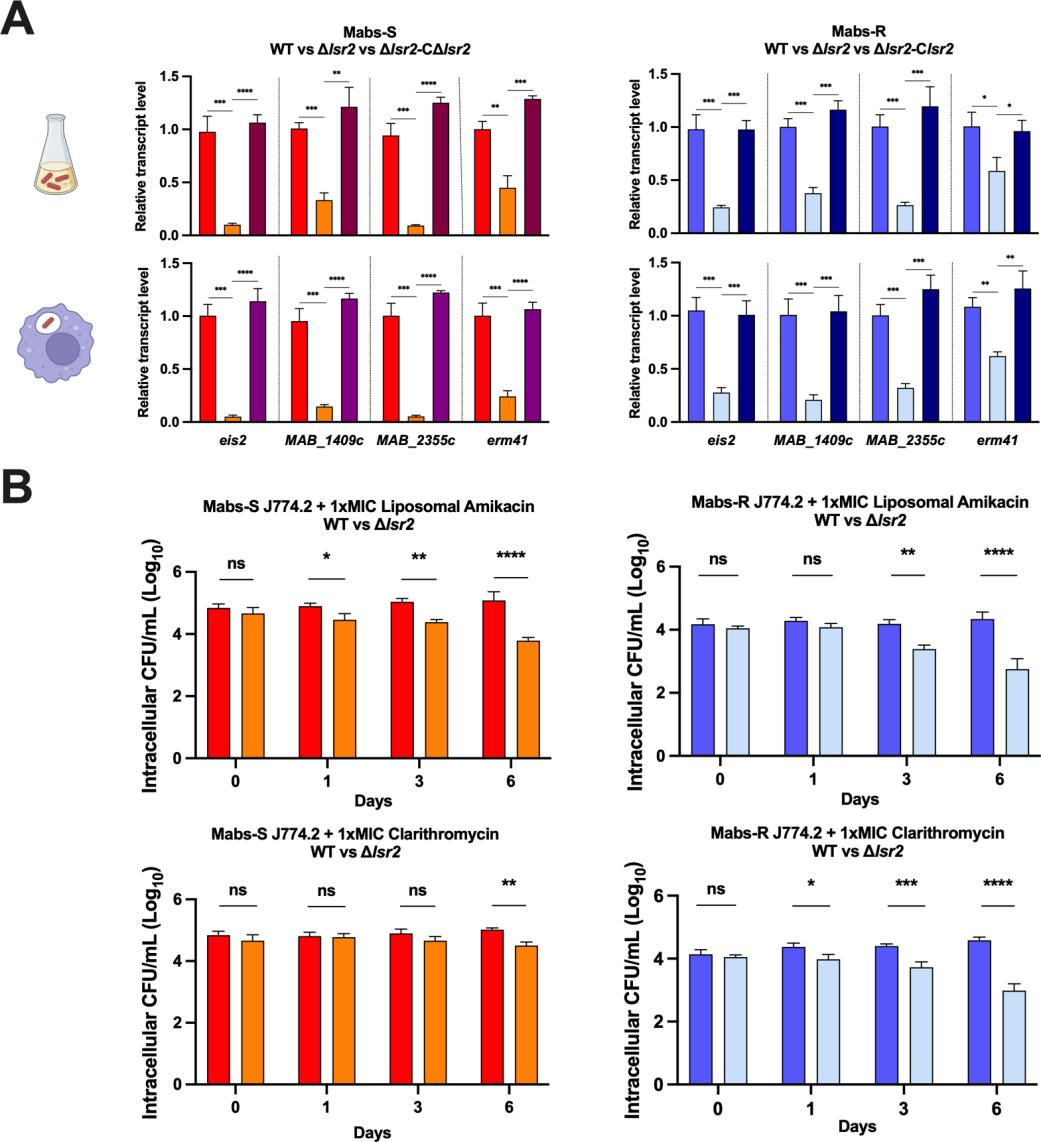
Enhanced sensitivity to aminoglycosides and macrolides due to diminished transcriptional expression of antimicrobial resistance genes in Mabs-S-Δ*lsr2* and Mabs-R-Δ*lsr2* strains. (**A**) Quantification of relative expression of antimicrobial resistance genes, *eis2, MAB_1409*c, *MAB_2355c, and erm41,* by RT-qPCR under planktonic growth conditions (upper side) and intracellular growth conditions (lower side) in Mabs-S-WT (red), Mabs-S-Δ*lsr2* (orange), Mabs-S-Δ*lsr2*-C*lsr2* (purple), Mabs-R-WT (blue), Mabs-R-Δ*lsr2* (light blue), and Mabs-R-Δ*lsr2*-C*lsr2* (dark blue). *sigA* transcript levels were used for normalization. For each assay, *n* = 3, and error bars are SEM. *P < 0.05, **P < 0.01, ***P < 0.001*,* and ****P < 0.0001 (Student’s *t* test). (**B**) Intracellular growth of *M. abscessus* strains: Mabs-S-WT (red), Mabs-S-Δ*lsr2* (orange), Mabs-R-WT (blue), and Mabs-R-Δ*lsr2* (light blue), following antibiotic treatments. Murine J774.2 macrophages were infected with mycobacteria at an MOI of 10 and subsequently treated with 1×MIC liposomal amikacin or 1×MIC clarithromycin. Intracellular growth post-antibiotic treatment was assessed by counting CFUs at various time points after treatment (days 0, 1, 3, and 6). The data are representative of three independent experiments and are presented as means ± SEM. Differences between means were analyzed by two-way ANOVA and the Tukey post-hoc test, allowing multiple comparisons. ns, non-significant, *P < 0.05, **P < 0.01, ***P < 0.001*,* and ****P < 0.0001*.*

Among the genes regulated by Lsr2 in both Mabs-S and Mabs-R, we have identified four genes (*eis2, MAB_1409c, MAB_2355*c, and *erm41*) encoding proteins modifying the activity of aminoglycoside and macrolide antibiotic families, both widely used in the clinical treatment of *M. abscessus* (9, 48–53). Our RNA-seq data show that Lsr2 activates these antimicrobial resistance genes, exhibiting higher expression in wild-type strains (Fig. 2A). Furthermore, RT-qPCR showed a significant decrease of expression of these four genes in Mabs-S-Δ*lsr2* and Mabs-R-Δ*lsr2* upon macrophage infections (Fig. 4A). Again, the wild-type expression level of these genes was restored by complementing the mutant strains with *lsr2*. To investigate whether this reduction in gene expression correlates with an increase in antibiotics sensitivity, we determined the minimum inhibitory concentrations (MICs) of amikacin, tobramycin, and clarithromycin for both wild-type and mutant strains (Table 1). Mabs-S-Δ*lsr2* and Mabs-R-Δ*lsr2* were more sensitive to one dilution factor for amikacin (4.5 and 8 µg/mL) and for tobramycin (4.5 and 5.5 µg/mL) compared to wild-type strains. For clarithromycin, Mabs-R-Δ*lsr2* (0.125 µg/mL) was only four times more sensitive than Mabs-R (0.5 µg/mL). Similar experiments performed after macrophage infections followed by treatments with 1×MIC liposomal amikacin and 1×MIC clarithromycin showed that both Mabs-S and Mabs-R respond more significantly to liposomal amikacin and clarithromycin treatments compared to the wild-type strains. For liposomal amikacin, this increased sensitivity is already significant on day 1 post-treatment for Mabs-S (*P <* 0.05) and day 3 for Mabs-R (*P <* 0.01). By day 6, both mutant strains exhibit a substantial difference in response to liposomal amikacin (*P <* 0.0001). Regarding clarithromycin, the impact of *lsr2* deletion on antibiotic response is particularly prominent in the Mabs-R-Δ*lsr2,* with a significant effect observed as early as day 1 post-treatment (*P <* 0.05) and a stronger response by day 6 (*P <* 0.0001) (Fig. 4B). These results strongly indicate that Lsr2, through its transcriptional effect, contributes in lowering susceptibility to antibiotics, thereby confirming its role in antibiotic resistance. Moreover, when assessing intracellular CFU counting following infection without antibiotic treatments, we observed a more important reduction in bacterial viability in the presence of antibiotics for *lsr2* mutant strains (Fig. S1).

**TABLE 1:** Increased sensitivity of *lsr2* mutant strains to aminoglycoside and macrolide antibiotics^*a*^

Antimicrobial agent		MIC (µg/mL) for			
		Mabs-S-WT	Mabs-S-Δ*lsr2*	Mabs-R-WT	Mabs-R-Δ*lsr2*
Aminoglycosides	Amikacin	9	4.5	15.5	8
	Tobramycin	9.5	4.5	11	5.5
Macrolides	Clarithromycin	0.5	0.5	0.5	0.125

^*a*^ Antibiotic susceptibility profiles (MICs) of *M. abscessus* wild-type and mutant (Δ*lsr2*) strains (μg/mL) after 3–5 days of incubation.

### DNA-binding profiles of Lsr2 on Mabs-S and Mabs-R genomes

**Fig 5:**
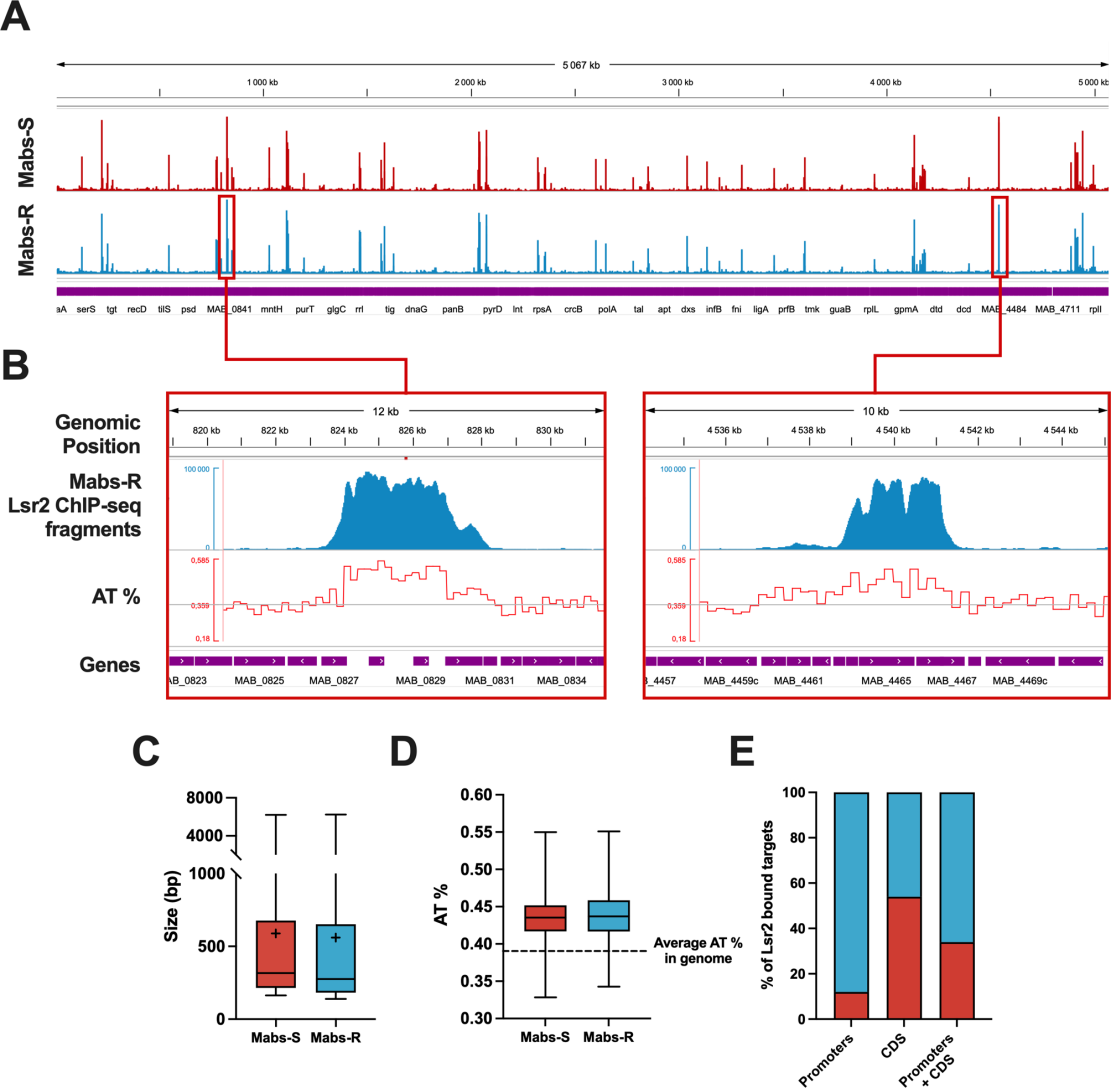
Modalities of Lsr2 binding on the *M. abscessus* genome. (A) Coverage of Lsr2 ChIP-seq fragments on the genomes of Mabs-S and Mabs-R morphotypes. (B) Distribution of Lsr2 ChIP-seq fragments is strongly correlated with enhanced AT content as exemplified for genomic regions encompassing genes from *MAB_0827* to *MAB_0842* and from *MAB_4463* to *MAB_4467*. The bottom part corresponds to gene positions. (C). Box plots showing the distribution of the lengths of binding regions of Lsr2 in the Mabs-S and Mabs-R morphotypes (with mean indicated as a « + »). (D) Box plots summarizing the preference of Lsr2 to bind to AT-rich regions of the *M. abscessus* Mabs-S and Mabs-R genome. (E) Histogram representing the percentage of Lsr2 within genomic features of operons (promoters, CDS, or promoters with CDS).

We used ChIP-seq to reveal how Lsr2 binding mode on *M. abscessus* genome can explain its effect on transcription regulation. Lsr2 binds around 10% of *M. abscessus* genes (511/4,920 genes in Mabs-S and 501/4,920 genes in Mabs-R), which is significantly less than what was observed in *M. tuberculosis* and *M. smegmatis* (respectively, 21% and 13%) (20). Peak calling analysis showed that Lsr2 binding profile is similar between Mabs-S and Mabs-R, with 348 enrichment peaks detected along the chromosome (Fig. 5A). Lsr2 binds to long tracts of DNA, forming extensive binding domains visualized through ChIP-seq as regions of enriched coverage that can span over 1 Kb and extend up to 6 Kb in certain regions (averages of 589 and 560 bp in Mabs-S and Mabs-R, respectively) (Fig. 5B and C). This ability to form long binding domains bears similarities to that observed in other NAPs such as H-NS in gram-negative bacteria and has also been described for Lsr2 *in vivo* in *M. tuberculosis* (24, 54, 55). Furthermore, we found that Lsr2 binds preferentially to AT-rich sequences (43% on average) (Fig. 5B and D; Fig. S2) compared to the average AT content observed in *M. abscessus* genome (35.9%) (7). Lsr2 had the same binding preference in other mycobacteria such as *M. tuberculosis* (20) and *M. smegmatis* (28). We next assess the distribution of Lsr2 over the different predicted operons constituting *M. abscessus’* genome. This analysis revealed that Lsr2 binds to 16% of the operons, with the ability to bind either operon promoters (12%), CDS (34%), or both operon promoters and CDS (54%) (Fig. 5E). These findings emphasize the preferential enrichment of Lsr2 on coding sequences, suggesting its direct role in gene regulation through extensive binding domains.

### Effects of Lsr2 binding on transcription regulation in *M. abscessus*

**Fig 6:**
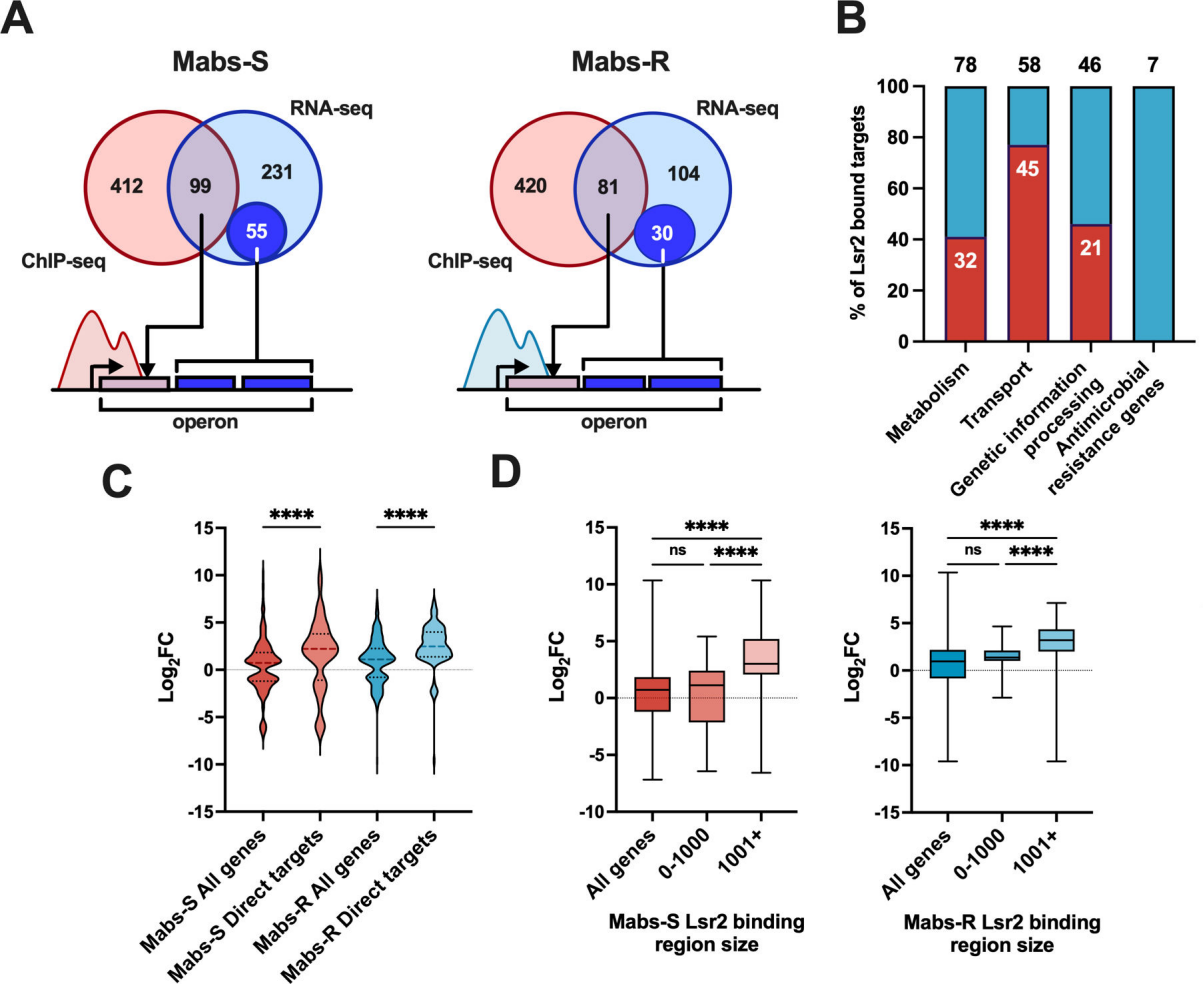
Integrative analysis RNA-seq/ChIP-seq. (**A**) Venn diagram showing the number of direct target genes of Lsr2, the number of genes regulated by Lsr2 without binding, and the number of genes bound by Lsr2 without regulatory effect in Mabs-S and Mabs-R. The numbers in the smallest circles show the number of regulated genes in the same operons as the direct targets. (**B**) Histogram representing the percentage of direct target genes of Lsr2 in the categories of metabolism, transport, genetic information processing, and antimicrobial resistance genes. The number of genes bound by Lsr2 and the total number of genes annotated in the KEGG database are also indicated on each histogram. (**C**) Violin plots comparing log_2_FC expression levels of all genes regulated by Lsr2 with those of direct target genes in Mabs-S and Mabs-R. (**D**) Box plots comparing the Log2FC expression levels of all genes regulated by Lsr2 with those of direct target genes, categorized by two domain size groups delineated by Lsr2, one with sizes less than 1 Kb and the other exceeding 1 Kb. Differences between means were analyzed by ordinary one-way ANOVA. ns, non-significant and ****P < 0.0001*.*

Integration of RNA-seq data with Lsr2 binding profiles shows that direct Lsr2 target genes constitute only 25% (99/385 genes) and 38% (81/215 genes) of all Lsr2 regulated genes in Mabs-S and Mabs-R, respectively. These numbers rise to 40% (154/385 genes) and 52% (111/215 genes) when considering the genes within Lsr2 bound operons that additionally exhibit altered expression in Lsr2 mutant strains (Fig. 6A). Secondly, although Lsr2 binds to a large number of genes or operons, many of them remain unregulated, indicating that its binding to DNA alone is insufficient to trigger transcription regulation. Indeed, 82% (422/511 genes) and 86% (433/501 genes) of the genes bound by Lsr2 are not differentially expressed in Mabs-S and Mabs-R mutants, respectively (Fig. 6A). If this lack of regulation can be explained by an absence of expression for a part of these genes in our experimental conditions, this result also suggests that unknown factors required for the transcriptional effect of Lsr2 are missing or that Lsr2 has only a structural effect on these genomic regions. Furthermore, among all the direct targets of Lsr2 (Fig. 6B), we found a stronger direct effect for Lsr2 in the regulation of genes involved in transport (77.5%), while all the antimicrobial resistance genes studied are indirectly regulated. To confirm the repressive function of Lsr2, we compared the expression levels between all Lsr2 regulated genes with its direct target genes in Mabs-S and Mabs-R. More than 74% of the direct target genes show significantly higher positive log_2_ fold change (log_2_FC) values compared to all Lsr2 regulated genes in both morphotypes, confirming the direct repressive role of Lsr2 in gene regulation (Fig. 6C). In order to assess the potential impact of the length of Lsr2 binding regions on its repressive effect, we investigated the distribution of expression levels among the direct target genes, categorized based on the size of the binding domains formed by Lsr2 (either less than 1 Kb or greater than 1 Kb). Significantly, more positive log_2_FC are correlated with binding domains larger than 1 Kb (*P <* 0.0001 in Mabs-S and *P <* 0.01 in Mabs-R), suggesting a positive correlation between the size of Lsr2 enriched domains and the repressive effect of Lsr2 (Fig. 6D).

## DISCUSSION

If NAPs are widely recognized as essential factors in the hierarchical structural organization of the bacterial chromosome, compelling evidence also highlights their crucial role in pathogen adaptation to changing environments, including their ability to thrive within host cells, such as macrophages, during infection. This includes reactive oxygen species (ROS) production, pH variations, hypoxia, or nutrient scarcity (56). The present study further establishes the pivotal role of NAP Lsr2 in the infectious strategy of *M. abscessus* as well as in its antibiotic resistance. Consistent with previous findings (29), our transcriptomic analysis confirms the impact of Lsr2 in controlling expression of key virulence factors on the intracellular and *in vivo* survival of *M. abscessus*. Among *lsr2* regulated genes, *eis2* is an important determinant of intracellular growth whose expression is highly induced in macrophages during infection. It controls the production of ROS, the sensitivity of bacteria to hydrogen peroxide (H_2_O_2_), and contributes to phagosomal membrane damage, thereby facilitating contact between the pathogen and cytosol (30). Similarly, *paa* genes that are upregulated by *lsr2* contribute to stress tolerance induced by antibiotics in *Acinetobacter baumannii* (57). These genes are also present in *Burkholderia cepacia* where the *paak*’s homolog is essential for the survival of the pathogen during infection in a rat model of chronic lung (58). The *sigD* and *sigE* genes, encoding two sigma factors regulated by Lsr2 in Mabs-S and Mabs-R, respectively, are involved in macrophage viability through resistance to oxidative stress. The loss of *sigD* reduces virulence, histopathology, and lethality of *M. tuberculosis* infection in mice (36–38). The KEGG analysis highlights a role of Lsr2 in the regulation of multiple metabolic pathways, with a particular emphasis for genes related to fatty acid degradation. This is intriguing, considering that during macrophage infections, a metabolic shift in carbon source utilization from sugars to lipids as a carbon source was previously observed (30), reflecting certain signatures of adaptation in intracellular life. Hence, the observed increase in mortality among *lsr2* mutant strains during infection (29) may be attributed to the challenges they face in adapting their metabolism in the absence of Lsr2. Likewise, Fe-S proteins, also regulated by Lsr2, are intracellular stress sensors and play a role in the genomic stability whose biosynthesis was induced in macrophages and amoeba by *M. abscessus* to adapt to an intracellular lifestyle (30). It has also been reported that impaired Fe-S protein biogenesis was deleterious to *Pseudomonas aeruginosa* growth (59) and decreases the ability of *Shigella flexneri* to invade epithelial cells lining the human colon (60).

Treatment of *M. abscessus* infection is particularly challenging, as this bacterium is resistant to most classes of antibiotics (9, 61). Here, our transcriptomic analysis revealed that four genes (*eis2, MAB_1409c, MAB_2355*c, and *erm41*) contributing to aminoglycoside and macrolide resistance are regulatory targets activated by Lsr2 in both morphotypes. *erm41,* encoding a methyltransferase, is widely studied for its role in inducible macrolide resistance in *M. abscessus,* observed in 40%–60% of clinical strains (49, 50, 62). *eis2* that codes for an N-acetyltransferase plays an important role in aminoglycoside resistance in *M. abscessus* by acetylating the first amino group of molecules like amikacin, in addition to its role in intracellular growth (53). The role of Lsr2 as a modulator of antibiotic resistance has been previously demonstrated in mycobacteria (63). In *M. smegmatis*, deletion of *lsr2* showed increased sensitivity of cells to nalidixic acid and rifampicin compared to the wild-type strain, in addition to better penetration for vancomycin through the cell wall (28). Similarly, in *M. abscessus*, we reported a greater sensitivity of *lsr2* mutant strains to liposomal amikacin and clarithromycin during macrophage infections and MIC determination. The increased sensitivity to antibiotics following *lsr2* deletion could also be explained by a deregulation of certain enzymes involved in the metabolism of lipids essential for cell wall formation, thereby modifying its permeability. Alternatively, the contribution of Lsr2 to antibiotic resistance in *M. tuberculosis* has been explained by the repression of an efflux pump for isoniazid following its overexpression (63). However, according to ChIP-seq results, these genes show no Lsr2 enrichment, suggesting their indirect regulation. Overall, the regulatory role of Lsr2 protein in response to antibiotics across various mycobacteria confirmed its potential as a target for the development of new antimicrobials.

Lsr2 was previously shown to bind to the promoter of the *mps* operon, while its deletion increases GPL level at the membrane of *M. smegmatis* (24, 47). However, we previously showed that the *lsr2* mutation did not restore the production of GPL in Mabs-R (29). These findings align with the identification of a CG insertion in the 5*'* region of the *mps1* gene, which completely abolishes the production of transcripts for the *mps1-mps2-gap* operon in this morphotype (17). Remarkably, our RNA-seq data demonstrated that *lsr2* deletion can restore transcripts elongation in this region to levels comparable to those observed in Mabs-S. Nevertheless, the presence of this indel is likely to result in a non-functional protein, thus preventing the bacteria from transitioning back to a smooth morphotype. This result still suggests an intriguing insight regarding Lsr2 functions in regulating transcription dynamics and RNA polymerase processivity, possibly via oligomerization or through changes in local DNA topology.

One of the fundamental goals of our study was to highlight the binding modes and to identify the direct target genes of Lsr2 on Mabs-S and Mabs-R genomes. Our ChIP-seq analysis identified 348 Lsr2 binding sites distributed throughout the *M. abscessus* chromosome. Like its ortholog H-NS, we have determined that Lsr2 presents a high affinity for AT-rich sequences. Previous reports suggest that Lsr2 and H-NS act as xenogeneic silencers for AT-rich regions acquired by horizontal gene transfer (21, 64, 65). In the case of *M. abscessus*, our findings do not support the binding of Lsr2 to these specific genes. Analysis of the Lsr2 genomic distribution in *M. tuberculosis* showed an enrichment pattern across 840 genes, including genes coding for type VII secretion systems of the ESX family, for the synthesis of cell wall lipids and PE/PPE proteins. In contrast, neither our ChIP-seq nor RNA-seq data revealed any binding or regulation by Lsr2 on genes involved in the secretion systems of *M. abscessus*. Our data also show that Lsr2 binding spans genomic regions that could sometimes exceed 1 Kb in size. As already suggested in previous works, this observation highlights the potential role of Lsr2 in repressing gene expression through a mechanism involving its oligomerization along promoters and coding sequences of its target genes. It has also been suggested that the oligomerization of Lsr2 plays a significant role in enhancing DNA stability by safeguarding it against nuclease degradation or ROS (66).

Finally, the existence of a limited repertoire of shared Lsr2 regulated genes between Mabs-S and Mabs-R first suggests differences in the Lsr2 distribution on the two morphotypes. Surprisingly, this distribution was strictly identical between both morphotypes with the same number of enrichment peaks detected. This result was further confirmed by a quantitative statistical analysis using the DiffBind R package. It has been reported that the mode of action of Lsr2 and H-NS could depend on additional features, such as the bridging of oligomers on distant DNA fragments, which most likely contribute to loop formation (24, 25). The different regulation between the two morphotypes as well as the presence of a large number of genes bound by Lsr2 without any effect on their transcription strongly suggests the intervention of other factors such as the existence of partner proteins or post-transcriptional modifications to explain the Lsr2 dependant transcriptional repression mechanism.

## MATERIALS AND METHODS

### *M. abscessus* strains, growth conditions, and DNA manipulations

Wild-type, *lsr2* mutant, and *lsr2* complemented strains of *Mycobacterium abscessus* 19977-IP, designated Mabs-S-WT, Mabs-R-WT, Mabs-S-Δ*lsr2*, Mabs-R-Δ*lsr2,* Mabs-S-Δ*lsr2*-C*lsr2,* and Mabs-R-Δ*lsr2*-C*lsr2,* were grown aerobically at 37°C in Middlebrook 7H9 broth or on Middlebrook 7H11 agar, supplemented with 2% glycerol, 1% glucose, and 0.05% Tween 80 when necessary. Construction of *lsr2* mutant and *lsr2* complemented strains was carried out during our previous study (29). Zeocin (25 µg/mL), kanamycin (250 µg/mL), and hygromycin (500 µg/mL) were added to cultures for strain selection. The Lsr2-FLAG-expressing strains used for ChIP-seq analysis, designated Mabs-S-Lsr2-FLAG and Mabs-R-Lsr2-FLAG, were obtained by cloning PCR-amplified homology regions surrounding the *lsr2* locus into a vector containing the *lsr2* gene sequence fused with three repeats of the FLAG epitope followed by a zeocin resistance cassette. Sequences of primers used for this construction are listed in Table S1. Replacement of the endogenous *lsr2* gene by *lsr2*-FLAG-zeocin sequence in Mabs-S and Mabs-R was performed as described previously (28, 55, 67). The insertion of the FLAG protein did not alter the phenotype of the bacteria nor did it affect the *in vitro* growth rates of the strains in liquid culture when compared to Mabs-S and Mabs-R (Fig. S3).

### RNA extraction, purification, and library preparation for RNA sequencing

Wild-type and *lsr2* mutant strains were grown until exponential growth phase with optical density value 600 nm (OD_600_) of 0.6 corresponding to approximately 2.4 × 10^8^ cells/mL. A total of 4.8 × 10^9^ cells were harvested, and total RNA from three biological replicates was extracted for each strain using the Monarch Total RNA Miniprep Kit (NEB). After DNase I treatment and elution, RNA concentrations were measured using the Qubit fluorometer (Promega). RNA integrity was evaluated using the Bioanalyzer system (Agilent), and all samples used for library preparation showed RNA integrity number scores >8. rRNA depletion was performed using the NEBNext rRNA Depletion Kit (Bacteria) (NEB), and the sequencing libraries were prepared using NEBNext Ultra II Directional RNA Library Prep Kit (NEB) following manufacturer’s instructions. RNA from *lsr2* complemented strains used for RT-qPCR experiments was extracted using the Monarch Total RNA Miniprep Kit (NEB).

### NGS sequencing and data analyses

All libraries were sequenced using a NextSeq500 (Illumina) at the Genomics platform of the UFR Sciences de la Santé (UVSQ, Montigny-le-Bretonneux, France). The reads were trimmed with fastp v0.23.2 (68) and mapped to the *M. abscessus* ATCC 19977 reference genome (NCBI accession number NC_010397) using STAR 2.7.6 a (69). Reads were counted with featureCounts v2.0 (70). Differential gene expression analysis was performed on R using the SARtools package using correction for batch effect (71). Significantly activated/repressed genes were selected with a threshold of 0.05 on the adjusted *P*-value and a log_2_ fold change of at least 1.

### Gene ontology term analysis

The gene ontology annotation of *M. abscessus* genome, specifically Biological Process and Molecular Function categories, was used to describe the roles and functions of the differentially expressed genes. The enrichment analysis was performed using the R package TopGO v.2.46.0 by combining the three different methods (*classic*, *elim,* and *weight*) with two statistical tests to evaluate the score significance (Fisher’s exact test and Kolmogorov-Smirnov test).

### ChIP-seq library preparation

Mabs-S-Lsr2-FLAG and Mabs-R-Lsr2-FLAG strains were grown until an OD of 0.6, and ChIP experiments were carried out in three biological replicates for each strain. Briefly, 50 mL of culture was fixed in 1% formaldehyde (Euromedex), quenched with glycine, washed with phosphate-buffered saline, and lysed using VK05 beads and Precellys grinder (three cycles: 8,700 rpm −3 × 20 s ON/60 s OFF, Bertin Technologies). For immunoprecipitation, 25 µg of chromatin was incubated with 25 µL of monoclonal anti-FLAG M2 magnetic beads (Sigma-Aldrich) on a rotary shaker for 16 hours at 4°C (29). The immunoprecipitated DNA and 1% of the total input were reverse crosslinked and eluted using the iPure v2 Kit (Diagenode). For library preparation, NEBNext Ultra II DNA Library Prep Kit for Illumina (NEB) was used. In parallel, ChIP-Seq libraries of Mabs-S and Mabs-R wild-type strains were prepared as a negative control. All the libraries were sequenced with paired ends (150 cycles) using an iSeq100 sequencer (Illumina).

### ChIP-seq data analysis

Reads were trimmed with fastp v0.23.2 (68), followed by mapping to the *M. abscessus* ATCC 19977 genome (NCBI accession number NC_010397) using Bowtie v2.4.5 (72) with a distribution of read coverages showing a mean of 35–58 as a proxy for sequencing depth. Fragments size of all libraries showed a distribution with median values ranging from 107 to 165 bp. PCR duplicates were marked by Picard 3.1.0 and further removed with samtools v1.15.1 (73). Peaks were visualized as raw sequencing coverage using the IGV genome browser (74) after conversion of the bam files in bigwig using Deeptools (75) without input normalization. Peak calling was performed using MACS v2.1.3.3 (76) with an FDR = 0.0029 and normalized against input libraries. Occurrence of Lsr2 peaks on operons or their promoters was detected using the intersect function of Bedtools v2.29.2 (77). The *M. abscessus* operons were predicted using Operon-mapper (78). Differential analysis of Lsr2 peaks between Mabs-S and Mabs-R was performed on R using the DiffBind package v3.10.0 (79).

### Drug susceptibility testing

MICs were determined for amikacin (1–256 µg/mL), tobramycin (0.25–64 µg/mL), and clarithromycin (0.06–32 µg/mL), according to Clinical and Laboratory Standards Institute guidelines (80). Stock solutions of amikacin (Mylan) and tobramycin (Mylan) were dissolved in water, and stock solutions of clarithromycin (Mylan) were dissolved in dimethyl sulfoxide. Drug susceptibility testing was determined using the microdilution method, cation-adjusted Mueller-Hinton broth in three biological replicates, as described previously (81, 82).

### Macrophage culture, infections, and intracellular survival after antibiotic treatments

Murine J774.2 macrophages were maintained at 37°C under 5% CO_2_ in Dulbecco’s modified Eagle medium (Gibco) supplemented with 10% fetal bovine serum (Gibco), penicillin (100 IU/mL), and streptomycin (100 µg/mL). Macrophages were seeded in a 24-well plate at a concentration of 5 × 10^4^ cells/mL of medium. Macrophage infections with wild-type and *lsr2* mutant strains at 10 MOI and treatments with 1×MIC liposomal amikacin (Insmed) and clarithromycin (Mylan) were carried out in three biological replicates, as described previously (16, 83, 84). The number of intracellular CFU per milliliter was determined on days 1, 3, and 6 post-infection and post-treatment. For day 0 plate, the number of intracellular CFU per milliliter was determined 3 hours after infection, without any antibiotic treatments. In parallel, the same infections were performed without antibiotic treatments as control for bacterial growth for three plates (days 1, 3, and 6).

### RNA extraction of mycobacteria from macrophage cocultures

Infections and RNA extractions were carried out in three biological replicates as described previously (30, 83). In brief, approximately 10^7^ cells of murine J774.2 macrophages were infected with wild-type, *lsr2* mutant, and *lsr2* complemented strains at 50 MOI. Macrophages were lysed 16 hours post-infection with a guanidine thiocyanate solution (4 M) plus β-mercaptoethanol, and total RNA was isolated from the bacterial pellets using TRIzol reagent (Ambion) in the presence of zirconia/silica beads. After bacterial cell disruption using a Precellys grinder (three cycles: 8,700 rpm − 3 × 20 s ON/60 s OFF, Bertin Technologies), RNA was extracted using chloroform isoamyl alcohol 24:1, precipitated with 0.8 volumes of cold isopropanol, washed with ethanol (70%), re-suspended in RNase-free water, and treated with the Turbo DNA-Free Kit (Invitrogen) to remove DNA contaminants.

### Reverse transcriptase quantitative PCR

cDNA were prepared using SuperScript III First-Strand Synthesis System Kit (Invitrogen). RT-qPCR were performed with a CFX96 thermal cycler (Bio-Rad), using the DyNAmo ColorFlash SYBR Green qPCR Kit (ThermoFisher), as described previously (14, 17). Target gene expression was quantified relative to the reference gene, *sigA*. The sequences of primers used for quantitative real-time PCR are listed in Table S1. Each RT-qPCR was performed on three biological replicates.

## Data Availability

All genomic data produced in the present project (RNA-seq and ChIP-seq) are available in the NCBI GEO database under accession number GSE239871.

## SUPPLEMENTAL MATERIAL

Supplemental material spectrum.03528-23-s0001.pdf Fig. S1 to S3; Tables S1 to S3.

ASM does not own the copyrights to Supplemental Material that may be linked to, or accessed through, an article. The authors have granted ASM a non-exclusive, world-wide license to publish the Supplemental Material files. Please contact the corresponding author directly for reuse.
