# Supplementary material for: Lsr2, a pleiotropic regulator at the core of the infectious strategy of Mycobacterium abscessus
Source: Microbiol Spectr. 2024 Feb 14;12(3):e03528-23. doi: 10.1128/spectrum.03528-23 (PMC10913753; doi:10.1128/spectrum.03528-23)
Supplement: Supplemental material — Fig. S1 to S3; Tables S1 to S3. [file spectrum.03528-23-s0001.pdf]

Supplementary data:

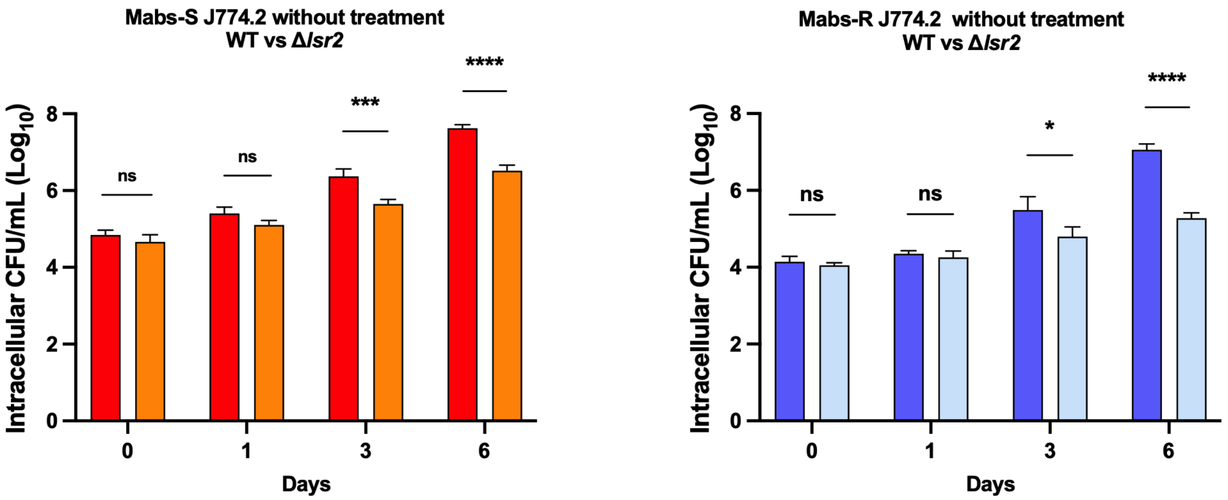

Figure S1: Intracellular growth of *M. abscessus* Mabs-S and Mabs-R *wild-type and lsr2* mutant strains in macrophages as control without antibiotics treatment. Murine J774.2 macrophages were infected with Mabs-S-WT (red), Mabs-S-Δ*lsr2* (orange), Mabs-R-WT (blue) and Mabs-R-Δ*lsr2* (light blue) at an MOI of 10. Intracellular growth was evaluated by counting CFUs at various time points post-infection (days 0, 1, 3, and 6). Data are representative of three independent experiments and represent means ± SEM. Differences between means were analyzed by two-way ANOVA and the Tukey post-test, allowing multiple comparisons. ns, non-significant, *$*P < 0.05$, ***$P < 0.001$, and ****$P < 0.0001$.*

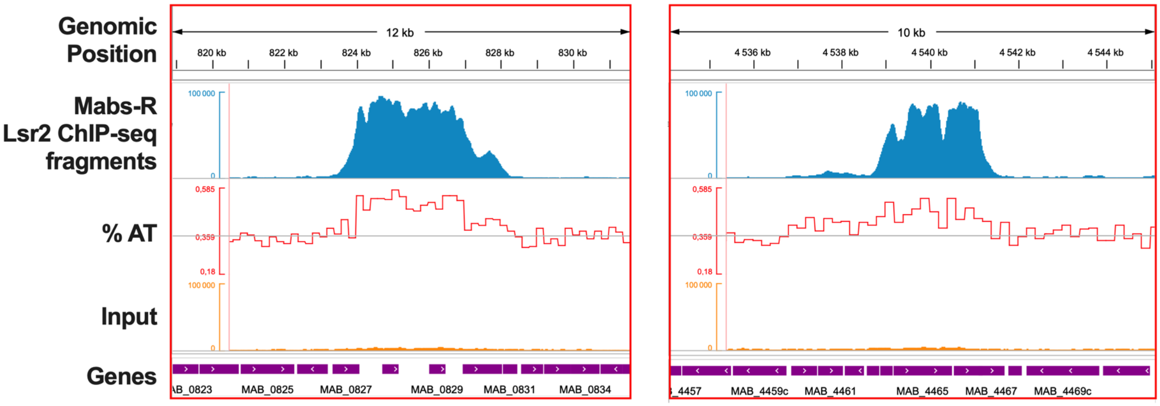

Figure S2: Lsr2 binds specifically on AT-rich sequences of the M. abscessus genome. Distribution of Lsr2 (in blue) is strongly correlated with enhanced AT content as exemplified for genomic regions encompassing genes from *MAB_0827* to *MAB_0842* and from *MAB_4463* to *MAB_4467*. Raw sequencing coverage from Input sample is also pictured in orange as a control for Lsr2 binding specificity. The bottom part corresponds to gene positions.

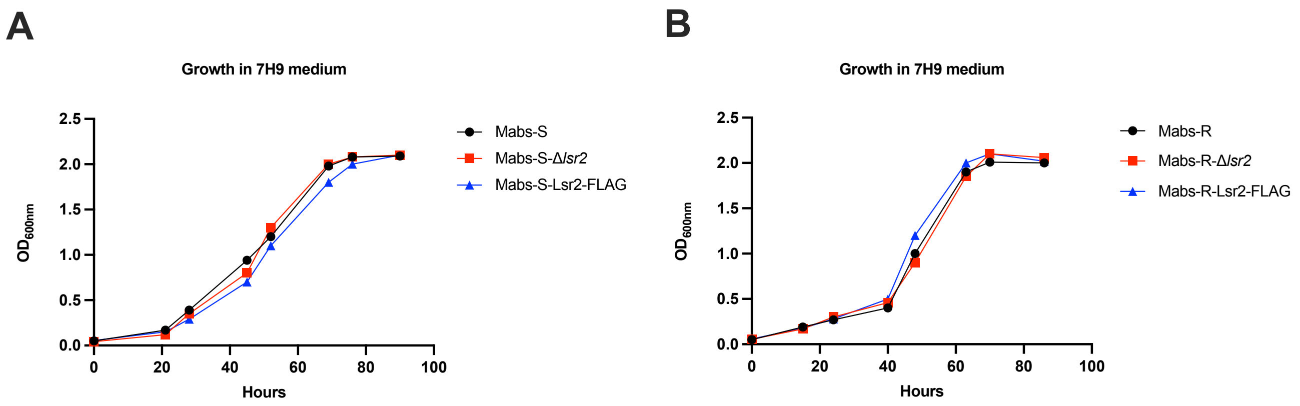

Figure S3: (A) Growth curve showing the absence of a growth defect in Mabs-S-Δ*lsr2* and Mabs-S-Lsr2-FLAG strains as compared to Mabs-S. **(B)** Growth curve showing the absence of a growth defect in Mabs-R-Δ*lsr2* and Mabs-R-Lsr2-FLAG strains as compared to Mabs-R.

Table S1. Primers used in this study

Name	Sequence
5'_Homology_Lsr2_region_Fwd	5'-aatgtatccgtcgttgtcagaag-3'
5'_Homology_Lsr2_region_Rev	5'-tcctttttccttccaaaagcg-3'
Lsr2-3XFLAG-zeocin_Fwd	5'- atggctaagaaggtcacgg-3'
Lsr2-3XFLAG-zeocin_Rev	5'-ggtctgacgctcagtggaac-3'
3'_Homology_Lsr2_region_Fwd	5'-tacgttcccgggacaatttc-3'
3'_Homology_Lsr2_region_Rev	5'-ctcgaaatcaccgcggtag-3'
SigA_Fwd	5'-tccgagaaagacaaggcttc-3'
SigA_Rev	5'-ccagctcgacttcctcttcg-3'
MAB_2355c_Fwd	5'-atgccaaactcgcgaacta-3'
MAB_2355c_Rev	5'-tctgccggtacatcaacacc-3'
erm41_Fwd	5'-ggaagatgtccggatagcgg-3'
erm41_Rev	5'-gtatcagtgcgctggtgact-3'
eis2_Fwd	5'-gtgttgtgagcgatgcgatc-3'
eis2_Rev	5'-cggaagaaagtcgtcggtca-3'
MAB_1409c_Fwd	5'-gtcgatcttctccgacgtcc-3'
MAB_1409c_Rev	5'-gtcatcgcaaggatcgggat-3'
lsr2_Fwd	5'-gagaccgttgaattcggtg-3'
lsr2_Rev	5'-gctgattacgcagcttctcc-3'
MmpL8_Fwd	5'-ctcgaatcagaccctgacgttcat-3'
MmpL8_Rev	5'-tgcccaacttggtgaatcccat-3'
MAB_2037_Fwd	5'-gcagacacgcatggcattaagt-3'
MAB_2037_Rev	5'-gaaaccgtagagcgacctgaaagt-3'

Table S2. Differentially expressed genes between Mabs-S and Mabs-R morphotypes of *M. abscessus*

Genes	Mab-S vs Mabs-R		Functions of encoded proteins
	Log_2FC	Adj. p-value	
mps1	-1.724	3.06E-03	Glycopeptidolipid biosynthesis protein
mps2	-3.881	6.15E-15	Glycopeptidolipid biosynthesis protein
gap	-2.633	4.79E-06	Integral membrane protein
MAB_2552c	-1.546	3.51E-15	Lipid transport and metabolism protein
MAB_4272c	2.477	0.005783288	Molecular chaperone GrpE (HSP-70 cofactor) protein
MAB_4273c	2.462	0.004734461	Molecular chaperone DnaK (HSP-70) protein
MAB_1242c	2.121	0.000254187	Hypothetical protein
MAB_1243c	1.875	0.005783288	Hypothetical protein
MAB_1247c	2.381	0.026330185	Hypothetical protein
nrdF	2.031	0.026330185	Nucleotide transport and metabolism protein
nrdE	1.894	0.005783288	Nucleotide transport and metabolism
nrdI	1.851	0.026330185	Nucleotide transport and metabolism
nrdH	1.677	0.034602339	Posttranslational modification protein

Table S3. Regulation of *mmpL8*$_{MAB}$ locus by Lsr2 in Mabs-S and Mabs-R morphotypes of *M. abscessus*

***mmpL8*$_{MAB}$ locus (genes)**	**Mabs-S *Δlsr2* vs WT**		**Mabs-S *Δlsr2* vs WT**	
	**Log_2FC**	**Adj. p-value**	**Log_2FC**	**Adj. p-value**
papA2	-6.362	6.08E-21	2.117	1.46E-02
lipP	-6.412	1.07E-23	2.046	1.15E-02
MAB_0854	-5.868	3.29E-18	1.949	2.60E-02
mmpL8	-5.886	7.64E-17	2.174	1.89E-02
tetR	-6.002	1.04E-06	4.656	2.27E-36
MAB_0857	-5.381	4.42E-20	4.342	2.25E-34
MAB_0858	-6.21	6.15E-50	1.323	5.60E-03
MAB_0859	-5.393	1.83E-27	1.041	1.76E-05
